# Improvement of psychometric properties of a scale measuring inpatient satisfaction with care: a better response rate and a reduction of the ceiling effect

**DOI:** 10.1186/1472-6963-7-197

**Published:** 2007-12-03

**Authors:** Leïla Moret, Jean-Michel Nguyen, Nathalie Pillet, Bruno Falissard, Pierre Lombrail, Isabelle Gasquet

**Affiliations:** 1 Inserm, U669, Paris, F-75014 France Univ. Paris-sud 11, Le Kremlin Bicêtre, F-94000 France; Univ. Paris 5, Paris, F-75015 France; AP-HP, Villejuif F-94804, France; 2 Public Health Department, University Hospital of Nantes, Nantes F-44093, France; 3 Direction de la Politique médicale, Assistance Publique – Hôpitaux de Paris, Paris, France

## Abstract

**Background:**

The objective was to solve two problems of an already validated scale measuring inpatient opinion on care: 1) a high non-response rate for some items due to the "not applicable" response option and 2) a skewed score distribution with high ceiling effect.

**Methods:**

The EQS-H scale ("échelle de qualité des soins en hospitalisation") comprised 26 items and 2 sub-scales of 13 items each, 'quality of medical information' (MI) and 'relationships with staff and daily routine' (RS). Three studies were conducted: a first mono-centre study (n = 552, response rate = 83.4%, self-completion of the scale the day before discharge) to construct a shorter version of the scale without the items with high non-response rate and maintaining those useful to ensure good internal validity (construct, convergent and divergent) and reliability; a second mono-centre study (n = 1246, response rate = 77.9%, self-completion of the scale before discharge) to confirm psychometric properties of the new version; a third multi-centre national study (n = 886, response rate 41.7%, self-completion at home 15 days after discharge) to test a new response pattern in order to reduce ceiling effect.

**Results:**

Six items having a non-response rate >20% were deleted, increasing rates of exhaustive response to all items from 15% to 48%. Factorial analysis supported the evidence for removing 4 more items to ensure good internal validity and reliability of the new version. These good results (initial variance explained: 43%; Cronbach's α: 0.80 (MI) and 0.81 (RS)) were confirmed by the second study. The new response format produced a normalisation of the 2 scores with a large decrease in ceiling effect (25% to 4% for MI subscale and 61% to 8% for RS). Psychometric properties of the final version were excellent: the 2 subscales (8 items each) explained 66% of the variance in principal component analysis, Cronbach's α were respectively 0.92 (MI) and 0.93 (RS).

**Conclusion:**

The new version of the EQS-H has better psychometric properties than the previous one. Rates of missing values are lower, and score distribution is normalized. An English version of this scale focused on quality of medical information delivered and on relationship with staff already exists, and this could be useful to conduct cross-cultural studies of health care service quality.

## Background

The assessment of satisfaction with care among hospitalized patients is increasingly recognized as a major component of quality management. Continuous quality improvement, comparison of hospital performances, and demands for accountability are some of the reasons that lead hospitals to measure patient satisfaction. Numerous studies on patient needs and expectations have been conducted [1-7]. According to Fitzpatrick [8] and others, patient satisfaction is a component of healthcare quality which reflects healthcare professionals' ability to meet their patients' needs and expectations. Donabedian [9] showed that the measurement of patient satisfaction is also part of the care provision process, and, as such, enables identification of dysfunction in the organization of care, and evaluation of efforts to improve quality. Several authors have found a relationship between patient satisfaction and clinical results [10-12]. Other studies, however, such as that by Barlesi [13] or by Vingerhoets [14] have not shown any impact of results of patient satisfaction surveys on the improvement of healthcare delivery. This issue of the use of results derived from measures of satisfaction as a tool to improve clinical performances has not been fully resolved, but authors agree that measurement is beneficial to patients (who are then viewed as partners in the care process) [15], and also to professionals (a confirmation of their professional skills) [16]. Finally, patient satisfaction is now one of the most common dimensions of performance on hospital dashboards. Patient satisfaction questionnaires have proliferated over the last decades as tools to measure health care from the patients' perspective. Nevertheless, in most cases, surveys have been criticized for their lack of a conceptual framework, and lack of valid and reliable instruments [17].

In France, measuring satisfaction has been mandatory since 1996 and several questionnaires have been developed over the last ten years [18-24]. Among the existing French scales, the EQS-H scale ('Echelle de Qualité des Soins en Hospitalisation') is used to assess inpatient satisfaction with medical information and relationships with staff. The validation of the first version of the scale was published in 1999 [19,20]. Although the EQS-H is convenient to use from the point of view of both hospitals and patients, its psychometric properties are compromised by a high rate of 'did not apply to me' responses (NA), analyzed as missing data, and by a skewed score distribution.

The main objective of this work was to optimize the psychometric properties of the scale by deleting items having a high rate of NA responses to increase scale stability, and by reducing ceiling effect to improve item response distribution. Overall, the aim was to make the questionnaire valid, reliable, easy to complete by all inpatients and suitable for quality of care improvement management.

## Methods

### The design of the research consisted in 3 studies

• Study A (scale shortening) to select items to be deleted on the basis of NA rates and using psychometric analysis combining Principal Component Analysis (PCA), convergent and discriminant validities, and reliability evaluated by Cronbach's α coefficients.

• Study B (replication phase) to confirm the psychometric properties of the new version of the EQS-H.

• Study C to test a new response pattern designed to reduce ceiling effect.

### Samples and study design (described in Table 1)

**Table 1:** Description of the designs of the studies

	**Study A**	**Study B**	**Study C**
**Main objective**	Scale shortening: to delete items with high non applicable response rate	Replication phase: to confirm internal structure of the new version	Test of a new response choice system to improve psychometric properties

**Date of study**	April 2002	June 2003	October 2004

**Number of participating centers**	1 teaching hospital	1 teaching hospital	12 hospitals (5 teaching, 3 general and 4 private)

**Inclusion criteria**	- French-speaking		
	- Able to complete a questionnaire		
	- Aged 18 and over		
	- Hospitalized full-time for at least 24 hours		
	- In medicine, obstetric and surgical units Eligible patients were included consecutively		

**Exclusion criteria**	- Language barrier		
	- Inability due to illness		
	- Children		
	- Refusal		
	- Outpatients		

**Number of questionnaires distributed**	662	1600	2125
**Too ill to participate**	87(13%)	245(15%)	
**Language barrier**	9(1.4%)	26(1.6%)	
**Refused to participate**	23(4%)	83(6%)	
**Number of respondents (Response rate, calculated from number of questionnaires distributed)**	552 (83.4%)	1246 (77.9%)	886 (41.7%)

**Mode of completion of the questionnaire (Q)**	- Q handed to patient by research assistant the day before discharge		- Q send by post 15 days after discharge at home with a prepaid envelope
	- Self-completion at hospital of the Q		- Self – completion of the Q
	- Q handed to the assistant in a sealed envelope		- Q sent back by mail using the prepaid envelope

**Version of EQS-H used**	26 items:	16 items:	16 items:
	- 13 for MI^1 ^subscale	- 9 for MI subscale	- 8 for MI subscale
	- 13 for RS^2 ^subscale 4 point Likert-scale*	- 7 for RS subscale 4 point Likert-scale*	- 8 for RS subscale 5 point Likert-scale**

Q: questionnaire

^1^MI subscale: quality of medical information

^2^RS subscale: relationship with staff and daily routine

*: Absolutely, nearly, not really, not at all.

**: Excellent, very good, good, moderate, poor

Studies A and B were mono-centre surveys carried out in the same conditions in April 2002 and in April 2003 in the teaching hospital of Nantes (France).

Study C was a multi-centre national survey conducted in October 2004 in 12 volunteering short-stay hospitals (teaching, general and private) taking part in a international performance assessment project co-ordinated by the World Health Organisation's pan-Europe project in Barcelona (PATH project: Performance Assessment Tools for Hospitals) [25]. Twenty hospital performance quality indicators were selected in several fields and a standardized evaluation of inpatient satisfaction was performed to assess the 'patient centeredness' dimension of the performance model.

### Questionnaire

The questionnaire used in Study A was the initial 26-item EQS-H comprising 2 sub-scales: "quality of medical information" (MI) (13 items) and "relationships with staff and daily routine" (RS) (13 items) [19]. In the validation study, the variance explained by the 2 factors was 42.3% and Cronbach's α was respectively for MI and RS subscales 0.88 and 0.87. Each item was rated from 1 (not at all) to 4 (absolutely), and a "NA" response was entered into analyses as a missing value. Only 15% of the patients responded exhaustively to all items in the validation study. The items of each sub-scale were summed, and then sums were rescaled to cover a range from 0 to 100 (the highest score reflecting the greatest satisfaction). Patient scores could be computed when at least half of the items plus one were completed.

The questionnaire used in Study B was the short version of the EQS-H (16 items).

The questionnaire used in study C was the 16-item EQS-H questionnaire constructed from studies A and B, with the response choice pattern modified from the previous 4-point format to a 5-point scale with 3 positive choices (excellent, very good, good) and 2 negative choices (moderate, poor). This format is considered to be the best way to avoid a ceiling effect, often highlighted in satisfaction questionnaires [26,27].

In line with previous studies and the literature [19,24], socio-demographic, medical and hospital-stay characteristics in relation to patient 16-item EQS-H scores were explored: gender, age, mode of admission, perceived health status compared to admission, perceived health status compared to people of the same age, satisfaction with life in general.

### Statistical analysis

*Study A: *Items with a 'NA' response rate higher than 20% were removed from the scale. An explanatory Principal Component Analysis (PCA) using a Varimax rotation on the correlation matrix was performed on the remaining 20 items. The number of factors was determined using the scree plot. Two criteria were used to attribute each item to one of the factors. First, a substantial loading on one principal component: like other authors [18], we chose coefficients >0.60 although the values generally accepted for the loadings are >0.40 [28]. Second, if an item loaded across several factors, it was attributed to the factor for which it maximized internal consistency measured by Cronbach's α. This strategy enabled removal of several items, for which neither a sufficient loading on principal components nor an adequate Cronbach's α could be obtained, yielding a robust shorter two-factor solution. The homogeneity of the dimensions was assessed using convergent validity (item correlations one with the other within a sub-scale greater than 0.40), and discriminant validity (correlation of items in one sub-scale with items in the other subscale less than 0.40) [29]. Correction for overlap was performed.

*Study B: *in order to confirm the internal validity and reliability of the 16-item EQS-H, we carried out a confirmatory PCA, convergent and discriminant analysis, calculation of Cronbach's α, and computed floor and ceiling effects.

*Study C: *the new format (5-Point scale) of the 16-item EQS-H scale was first compared to the initial response scale in terms of psychometric properties, mean scores, floor and ceiling effects. A two-factor solution confirmatory PCA was performed using a Varimax rotation on the correlation matrix. Two criteria were used to attribute each item to one of the factors: a substantial loading (>0.60) on one principal component, or, if an item loaded across several factors, it was attributed to the factor for which it maximized internal consistency measured by Cronbach's α. Convergent and discriminant validities were obtained, correcting for overlap.

Structural Equation Modelling (SEM) was performed to confirm factorial structure. SEM is a generalization of linear regression and factor analysis models [30,31]. These models provide the simultaneous estimation of several multiple linear regressions. Variables in the regressions can be observed or latent. The latent variables are considered to be an underlying common factor that explains the pattern of correlations observed in the group of observed variables [32]. Several statistical indices enable verification of model fit and selection of the best-suited model. Since this statistical technique can prove to be unstable, it is recommended that several be used in order to choose the model that maximises certain criteria. The main indicators used for this are the RMSEA (Steiger's Root Mean Square Error of Approximation), the fit being considered good if <0.1 and very good if <0.05, the NFI (Bentler and Bonnet's Normed Fit Index), considered as good if >0.95, and the GFI (Goodness of Fit Index), considered as good if >0.85 [33,34].

Finally, a general multivariate linear model was used to adjust the 16-item EQS-H global score on socio-demographic variables.

All study analyses were conducted using SPSS software (version 11) and SPAD. SEM was performed using SAS 8.2 and "PROC CALIS" procedure.

## Results

### Scale shortening procedure (Study A)

Six items had a frequency of 'NA' response higher than 20%. Four of them were related to patient autonomy: help for psychological problems (response rate = 23.0%), help with meals (48.6%) help with washing (33.9%), help with going to the toilet (38.0%), and 2 items concerned patients' relatives: involvement in information sessions with relatives (46.6%), information given to relatives (33.3%). These 6 items were the first to be removed in order to decrease the rate of missing values.

An explanatory PCA on the remaining 20 items made it possible to identify 2 dimensions based on the scree plot. The first two eigenvalues were 5.03 for the first component and 2.68 for the second. Four more items were removed: one item related to obtaining answers from doctors loaded on the two factors (0.36 and 0.38 respectively) and did not maximize Cronbach's α coefficient; one item concerning involvement in discharge from hospital was correlated to the 'MI' subscale in the 26-item EQS-H but had a low loading with the new 'MI' subscale (0.26) and did not maximize Cronbach's α coefficient. Lastly, Cronbach's α increased from 0.72 to 0.76 in the subscale 'RS' when two items concerning bedside behaviours on the part of staff and doctors were eliminated.

Finally, the short EQS-H scale comprised 16 items. Forty-eight percent of patients answered the items exhaustively (versus 15% in the initial 26-item EQS-H scale). An explanatory PCA showed a robust two-factor solution: 'MI' (9 items) and 'RS' (7 items) accounting for 42.1% of the variance (Table 1). Cronbach's α coefficients were respectively 0.83 and 0.82. Moreover, correlations between items within a given subscale were all higher than 0.40 and correlations between items and those of the other sub-scale were lower than 0.40.

### Replication phase (Study B)

A confirmatory PCA on the two factors confirmed all the results obtained. The two-factor solution: 'MI' (9 items) and 'RS' (7 items) accounting for 42.9% of the variance (Table 2). All items had a very good loading on their own factor except the first item of the second factor (0.38) ('I could identify the doctor in charge of me') (Table 2). Cronbach's α coefficients were close to the first obtained (0.80 and 0.81 respectively). Convergent and discriminant validities confirmed the consistency of the 2 sub-scales.

**Table 2:** Results of PCA using varimax rotation in the 3 studies

		**Study A (n = 552)**		**Study B (n = 1246)**		**Study C (n = 886)**	
	Factor	RS^2^	MI^1^	RS^2^	MI^1^	RS^2^	MI^1^

	% of explained variance	21.5	20.6	20.4	22.5	54.5	11.0

**Abbreviation**	**I received clear information about: **						

Info1	- symptoms	*0.25*	***0.63***	*0.10*	***0.61***	*0.22*	***0.79***
Info2	- the purpose of the tests	*0.08*	***0.57***	*0.07*	***0.57***	*0.27*	***0.77***
Info3	- the results of the tests	*0.06*	***0.65***	*0.12*	***0.63***	*0.23*	***0.81***
Info4	- purpose of the treatments	*0.06*	***0.70***	*0.18*	***0.65***	*0.28*	***0.78***
Info5	- the possible side-effects of these treatments	*0.04*	***0.65***	*0.13*	***0.66***	*0.24*	***0.76***
Info6	- warning signs to look for	*0.06*	***0.73***	*-0.03*	***0.71***	*0.37*	***0.67***
Info7	- when to resume activities after discharge	*0.05*	***0.71***	*0.09*	***0.68***	*0.34*	***0.71***
Info8	- medical follow-up	*0.05*	***0.66***	*-0.01*	***0.67***	*0.39*	***0.62***

							

Rela1	I could identify the doctor in charge of me	*0.11*	***0.51***	*0.10*	***0.38***	***0.55***	*0.44*
Rela2	There was enough privacy during medical care	***0.66***	*0.07*	***0.65***	*0.16*	***0.65***	*0.35*
Rela3	I received enough help in my daily routine	***0.78***	*0.09*	***0.78***	*0.09*	***0.79***	*0.30*
Rela4	Everything possible was done to relieve my pain	***0.77***	*0.06*	***0.71***	*0.08*	***0.69***	*0.34*
Rela5	I saw nurses as often as I wished	***0.49***	*0.05*	***0.51***	*0.02*	***0.81***	*0.30*
Rela6	There was good co-ordination in the department	***0.76***	*0.05*	***0.78***	*0.12*	***0.84***	*0.27*
Rela7	There was a good atmosphere in the department	***0.57***	*0.15*	***0.56***	*0.09*	***0.84***	*0.23*
Rela8	The nurses were fully available	***0.69***	*0.10*	***0.66***	*0.09*	***0.85***	*0.23*

^1^MI subscale: quality of medical information

^2^RS subscale: relationship with staff and daily routine

However, the ceiling effect remained high, at 24.7% for MI dimension and 61.2% for RS dimension (Table 3).

**Table 3:** Comparison of the psychometric properties of the 2 formats of the 16-item EQS-H (Study B – 4-point Likert scale and Study C – 5-point Likert scale)

	**Study B 4-point Likert scale (N = 1246)**			**Study C 5-point Likert scale (N = 886)**		
**Items properties**	**MI**^1^	**RS**^2^	**Global**	**MI**^1^	**RS**^2^	**Global**

# of items in the scale	9	7	16	8	8	16
% of questionnaires with at least 1/2 the items completed	96.2	100.0	100.0	96.2	99.2	98.6
# of items with "Missing data" >20%	1	0	1	0	0	0
# of items with "Does not apply" response >20%	1	0	1	0	0	0
# of items with ceiling effect >50%	7	7	14	0	0	0
# of items with floor effect >50%	0	0	0	0	0	0

**Scaling properties**						

Mean score (+-SD)	79.9 (20.8)	93.6 (11.4)	-	59.2 (21.0)	69.0 (19.8)	64.2 (18.7)
Skewness value/SE	-1.23/0.07	-2.41/0.07	-	-0.08/0.08	-0.23/0.08	-0.07/0.08
Median	85.7	100	-	59.4	68.8	64.1
Ceiling effect (%)	24.7	61.2	-	4.3	8.4	2.6
Floor effect (%)	0.5	0	-	0.4	0	0
Inter-scale correlation	0.26	-	-	0.67	-	-
# of item correlation with own scale >0.40	9	7	-	8	8	-
# of item correlation with own scale greater than with other scales	9	7	-	8	8	-
Cronbach's αgoefficient	0.80	0.81	-	0.92	0.93	0.95
Sum of square of the factors before rotation				32.5%	33.0%	65.5%
% of variance explained by the factor	22%	21%	43%	11.0%	54.5%	65.5%

^1^MI subscale: quality of medical information

^2^RS subscale: relationships with staff and daily routine

### Testing a new response pattern (Study C)

#### Floor and ceiling effects

The new response format associated with a 5-point scale yielded a very marked decrease in ceiling effect accompanied by a normalisation of the scores (Table 3). Mean scores were respectively 59.2 (SD = 21.0) for 'MI' and 69.0 (SD = 19.8) for 'RS', close to the median (59.4 and 68.8). Skewness values were between -0.48 and -0.05. Only 4.3% and 8.4% of patients respectively still obtained a score of 100.

#### Psychometric properties of the final scale

The confirmatory two-factor PCA rotated using the Varimax procedure accounted for 65.5% of the variance for the 2 first principal components (54.5% for the first factor 'RS'(8 items) – eigenvalue: 8.73, and 11.0% for the second 'MI' (8 items) – eigenvalue: 1.77) (Tables 2 and 3). One item on the ability to recognize the doctor in charge loaded on "MI" factor, in contrast to the previous result. 'Cronbach's α coefficients were excellent: respectively 0.92 for 'MI', 0.93 for 'RS' and 0.95 for the 16-item EQS-H scale overall. Convergent and discriminant validity were good, all items had a correlation >0.40 with their own subscale, and correlations between items and those of the other sub-scale were lower than 0.40. Inter-subscale correlation was 0.67 (Table 3).

**Figure 1:**
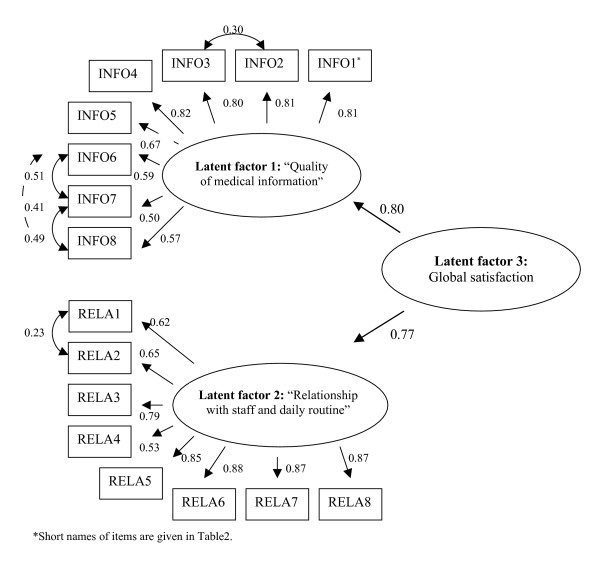
Structural equation model of the new version of EQS-H (N = 793). *Short names of items are given in Table 2.

Structural Equation Modelling confirmed the existence of 2 latent factors ('MI' and 'RS') but the best characteristics were obtained with a hierarchical model including the 2 latent factors and a global satisfaction latent factor, bringing the 16 items together (Figure 1). Goodness of fit of the data was very good with RMSEA = 0.063, NFI = 0.954 and GFI = 0.943. All the structural coefficients were significant (p < 0.001).

The 16-item EQS-H overall score was associated with several adjustment variables in a general multivariate linear model. Scores were significantly higher for males (p = 0.019), for older patients up to 65 years (p = 0.002), for those who thought they had better health than people of the same age (p < 0.001), for those who thought they had better health status compared to admission day (p < 0.0001) and for patients who were more satisfied with their life in general (p < 0.0001).

## Discussion

These three studies, carried out on large samples of subjects, made it possible to significantly improve the psychometric properties of the previously validated inpatient satisfaction scale EQS-H. The validation process demonstrated high added value after reducing and modifying the questionnaire, and the new form appears to be valid and reliable, and to contribute to the non-biased subjective evaluation of in-patient reported outcomes.

The shortening and validation strategies presented here follow most of the recommendations of 'good practice' for satisfaction scale validation [11,35]. To begin with, our strategy was to promote higher response rates. Six items were initially removed from the scale, on account of a high rate of 'NA' responses. The EQS-H is an in-patient global satisfaction questionnaire, which should be applicable to most patients admitted to hospital units, whatever their autonomy. Badly impaired autonomy only concerns a few patients so that items relating to this aspect may not be relevant to the large majority of subjects. Reducing the length of the questionnaire, which involved some of these items, increased exhaustive response completion threefold. Low response rates, while entailing loss of data, can also introduce bias into survey findings because non-respondents may differ from respondents in ways that affect their evaluation of different aspects of care. As recommended by Coste [35], the development of the short EQS-H scale complied with two successive phases: the shortening process itself, which was performed via study A, and the validation process, conducted independently on another large sample of subjects (Study B). The replication phase strongly confirmed our findings.

Secondly, the objective was to reduce the ceiling effect highlighted in initial EQS-H scale in order to normalize the distribution curve. As suggested by Streiner and Ware [26,36], we modified responses choices from a 4-point scale to a 5-point scale with 3 positive choices and no neutral (median) response choice. Patients who took part in the studies were generally highly satisfied with the quality of care [37] and these modifications in the response format provide better sensitivity. The normalization of the distribution also made it possible to improve the statistical validity of comparisons and to obtain better results in satisfaction score modelling when adjusted variables are tested. Following response pattern alterations, the ceiling effect disappeared.

Finally, the new EQS-H questionnaire is a self-report instrument comprising 16 items, covering two very important domains of patient satisfaction, 'Quality of medical information' (8 items) and 'Relationship with staff' (8 items). These two factors are related to interpersonal aspects of care, which are both predictors of patient opinion on care [38]. Donabedian emphasized that "the interpersonal process is the vehicle by which technical care is implemented and on which its success depends" [39]. There is consistent evidence across settings that the most important health service factor affecting satisfaction is the patient-practitioner relationship, including their primary role in information provision [4,27]. Patient information has become crucial in health care because it is essential to enable the patient to take part, freely and in an enlightened manner, in medical decisions and resulting care provision.

Our results support good content, construct and concurrent validity for the new version of the measure. The new version of the EQS-H demonstrated excellent internal consistency (over 0.90). Items had strong loadings on the two factors identified by PCA and accounted for more than 65% of the variance. Convergent and discriminant validity were good. Concurrent validity was excellent. Socio-demographic variables related to scores are those usually described in literature. To confirm our results, Structural Equation Modelling was performed, and this strongly supports the possibility of calculating a global satisfaction score. The fact that high correlations exist between all these items and factors is not surprising, and helps to explain why the item related to the identification of the doctor correlates highly with the MI dimension in the two first studies and with the RS dimension in the last.

Nevertheless, this work entails several limitations: half of the questionnaires systematically present more than one missing value. Information concerning relatives is no longer explored. Professional help received in daily routine is limited to 2 items instead of 6. The 2 remaining items related to patient autonomy are the most important ones (pain relief and help for daily routine). However, depending on patient samples studied, these items could be part of the questionnaire but not be taken into account in the scoring. The response rate for the postal study was around 40% and it is possible that the representativeness of the sample could be biased, due to the loss of data from the non-respondents, although most authors accept this rate of response and consider that non-respondents are generally shown not to be significantly different from respondents in terms of satisfaction scores [20].

## Conclusion

This work emphasizes the need to check and to refine psychometric properties of the questionnaires previously developed. The EQS-H is one of the well-known scales very often used to assess inpatient satisfaction with quality of medical and nursing care within hospitals. Items are clinically relevant in hospital setting and promoting its use in different inpatient clinical settings is already planned in our hospital in order to increase the usefulness of the tool for clinicians. After issue of a summary of results to both teams involved, highlighting priorities for improvement efforts, hospital staff screened areas for further investigations and substantial improvements were noted in several units, concerning such issues as privacy, pain and amount of patient information. However, the diffusion of the questionnaire does need to be backed up by a communication campaign, because results from patient satisfaction surveys aiming to improve healthcare delivery are still frequently under-used by healthcare teams and not widely publicised [40]. The actual impact of any corrective action taken on patient satisfaction in hospital has not been a consistent finding [41,42]. Taking concrete action for improvement, for instance circulating informative documents or establishing the traceability of the information chain, seems easier than actually changing behaviours among healthcare professionals [42]. Finally, given the instrument's good psychometric properties as revealed in this study, further work is needed to confirm the excellent validity and reliability obtained. Complementary analyses using item response models to study the difficulty of items and their homogeneity in relation to the rest of the questionnaire could be useful, as could differential item functioning analyses, so as to study scores not solely as overall averages but also according to sub-groups, for instance healthcare departments, medical specialities or case-mixes. In addition, the dimensions explored by the EQS-H are not limited to the French healthcare system, and further scale validation in other countries and cultures is required, since it would facilitate cross-cultural studies of health care service quality. English, Spanish and Italian versions of the EQS-H satisfaction scale are already available (see in Additional file 1 English free access version of the questionnaire).

## Pre-publication history

The pre-publication history for this paper can be accessed here:



## Supplementary Material

Additional File 1 EQS-H questionnaire: English version. The file presents the English free access version of the questionnaire Click here for file
