# Supplementary material for: Improvement of psychometric properties of a scale measuring inpatient satisfaction with care: a better response rate and a reduction of the ceiling effect
Source: BMC Health Serv Res. 2007 Dec 3;7:197. doi: 10.1186/1472-6963-7-197 (PMC2225402; doi:10.1186/1472-6963-7-197)
Supplement: Additional File 1 — EQS-H questionnaire: English version. The file presents the English free access version of the questionnaire [file 1472-6963-7-197-S1.doc]

**EQS-H questionnaire: English version**

Dear Sir or Madam

You were recently admitted to (….) Hospital and we would like to know how satisfied you were with the way in which your needs were catered for while you were there.

Below you will find some statements that people may make about their hospital experience..

For each statement, please tick the box that best corresponds to your experience. Your answers will remain confidential and strictly anonymous. Please return the questionnaire, whether completed or not, in the prepaid envelope provided.

Thank you for taking part.

The research team

**Information given to me in this hospital about my condition and treatment**

**How clear were the explanations?**

- about my symptoms:

Poor Average Good Very good Excellent

- about why I needed certain examinations or tests:

Poor Average Good Very good Excellent

- about the results of my examinations or tests:

Poor Average Good Very good Excellent

- about the aims of my treatment (medication, operation, etc):

Poor Average Good Very good Excellent

- about possible side effects of my treatment:

Poor Average Good Very good Excellent

### The hospital staff and the ward

**How satisfied were you with the following?**

- the information as to which doctor was in charge of me:

Poor Average Good Very good Excellent

- efforts to ensure my privacy:

Poor Average Good Very good Excellent

- assistance given me for day-to-day activities (eating, washing, dressing etc):

Poor Average Good Very good Excellent

- assistance for pain relief:

Poor Average Good Very good Excellent

- the promptness of nurses in coming when called:

Poor Average Good Very good Excellent

# Please turn to next page

- the organisation of the ward:

Poor Average Good Very good Excellent

- the atmosphere in the ward s

Poor Average Good Very good Excellent

- the readiness of nurses to spend time with me s

Poor Average Good Very good Excellent

### Information given to me when leaving the hospital

**How clear were the explanations?**

- about the symptoms I should watch for in the future:

Poor Average Good Very good Excellent

-about the activities I could resume after discharge (job, sport, etc):

Poor Average Good Very good Excellent

about my medical care after discharge:

Poor Average Good Very good Excellent

### Your overall opinion

I had as much say as I wanted in medical decisions that concerned me

Very rarely or never Sometimes Often Nearly always Always

On the whole, the care and treatment that I received was

Poor Average Good Very good Excellent

### Details about you and your health

Male /_/ Female /_/

Age /_/_/_/

Was your stay in hospital planned ?

YES, it was planned NO, I was admitted in emergency

Did your health condition improve in the course of your hospital stay ?

NO /_/ YES, a little /_/ YES, a lot /_/

Compared to other people of your age, how do you view your health ?

Worse than others of my age /_/ About the same as others of my age /_/ Better than others of my age/_/

How satisfied are you with your life IN GENERAL (disregarding your stay in hospital)?

Tick the appropriate box between 1 for “not at all satisfied” and 7 for “completely satisfied”.

1 2 3 4 5 6 7

In your opinion, what would be the priorities for improvement in this hospital?

……………………………………………………………………………………………

Please return this questionnaire in the prepaid envelope provided.

Thank you for answering the questionnaire.
